# 1-Methyl-1-azonia-3,5-diaza-7-phosphatricyclo­[3.3.1.1^3,7^]decane tetra­fluoro­borate

**DOI:** 10.1107/S1600536808003401

**Published:** 2008-02-06

**Authors:** Piotr Smoleński, Alexander M. Kirillov, M. Fátima C. Guedes da Silva, Armando J. L. Pombeiro

**Affiliations:** a Centro de Química Estrutural, Complexo Interdisciplinar, Instituto Superior Técnico, TU Lisbon, Av. Rovisco Pais, 1049-001 Lisbon, Portugal; b Universidade Lusófona de Humanidades e Tecnologias, ULHT Lisbon, Av. do Campo Grande, 376, 1749-024, Lisbon, Portugal

## Abstract

The title compound, C_7_H_15_N_3_P^+^·BF_4_^−^ or [PTA-Me][BF_4_], is the *N*-methyl­ated derivative of the well known water-soluble amino­phosphine 1,3,5-triaza-7-phosphaadamantane (PTA). The asymmetric unit consists of a cage-like cation [PTA-Me]^+^ and a disordered tetra­fluoro­borate anion; two F atoms are disordered equally over two sites. A network of weak inter­molecular C—H⋯F hydrogen bonds results in a three-dimensional supra­molecular assembly.

## Related literature

For general background, see: Kirillov *et al.* (2007 ▶); Smoleński & Pombeiro (2008 ▶). For a comprehensive review of PTA chemistry, see: Phillips *et al.* (2004 ▶). For the synthesis of PTA and [PTA-Me]I, see: Daigle *et al.* (1974 ▶); Daigle (1998 ▶). For related organic structures, see: Jogun *et al.* (1978 ▶); Forward *et al.* (1996 ▶); Otto *et al.* (2005 ▶); Kirillov *et al.* (2008 ▶). For related metal–organic structures, see: Kovacs *et al.* (2004 ▶); Smoleński *et al.* (2003 ▶); Pruchnik *et al.* (1999 ▶).
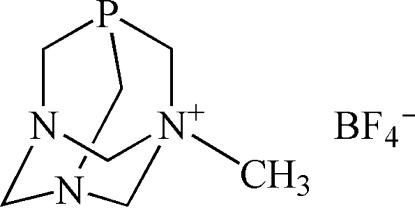


## Experimental

### 

#### Crystal data

C_7_H_15_N_3_P^+^·BF_4_^−^
*M* *_r_* = 259.00
Orthorhombic, 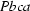

*a* = 11.994 (2) Å
*b* = 11.6933 (18) Å
*c* = 15.569 (2) Å
*V* = 2183.5 (6) Å^3^
*Z* = 8
Mo *K*α radiation
μ = 0.28 mm^−1^
*T* = 150 (2) K
0.16 × 0.12 × 0.10 mm

#### Data collection

Bruker SMART CCD diffractometer
Absorption correction: multi-scan (*SADABS*; Bruker, 2004 ▶) *T*_min_ = 0.956, *T*_max_ = 0.972
10402 measured reflections
1948 independent reflections
1391 reflections with *I* > 2σ(*I*)
*R*_int_ = 0.061

#### Refinement

*R*[*F* ^2^ > 2σ(*F* ^2^)] = 0.045
*wR*(*F* ^2^) = 0.121
*S* = 1.05
1948 reflections
164 parameters
H-atom parameters constrained
Δρ_max_ = 0.55 e Å^−3^
Δρ_min_ = −0.36 e Å^−3^

### 

Data collection: *SMART* (Bruker, 2004 ▶); cell refinement: *SAINT* (Bruker, 2004 ▶); data reduction: *SAINT*; program(s) used to solve structure: *SIR97* (Altomare *et al.*, 1999 ▶); program(s) used to refine structure: *SHELXL97* (Sheldrick, 2008 ▶); molecular graphics: *Mercury* (Macrae *et al.*, 2006 ▶); software used to prepare material for publication: *SHELXL97*.

## Supplementary Material

Crystal structure: contains datablocks I, global. DOI: 10.1107/S1600536808003401/hb2695sup1.cif

Structure factors: contains datablocks I. DOI: 10.1107/S1600536808003401/hb2695Isup2.hkl

Additional supplementary materials:  crystallographic information; 3D view; checkCIF report

## Figures and Tables

**Table 1:** Hydrogen-bond geometry (Å, °)

*D*—H⋯*A*	*D*—H	H⋯*A*	*D*⋯*A*	*D*—H⋯*A*
C2—H2*A*⋯F2^i^	0.99	2.54	3.438 (3)	151
C5—H5*A*⋯F4^ii^	0.99	2.35	3.314 (3)	166
C6—H6*B*⋯F4	0.99	2.46	3.364 (3)	152
C11—H11*B*⋯F2^iii^	0.98	2.43	3.350 (4)	156

Symmetry codes: (i) 
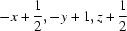
; (ii) 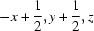; (iii) 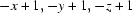.

**Fig. 1.**
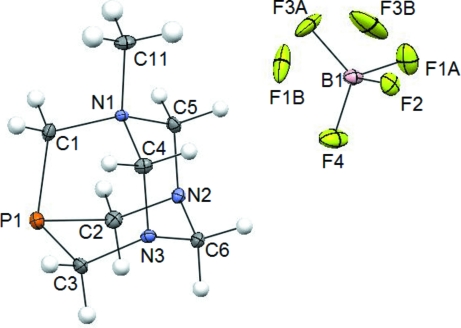
The molecular structure of (I) with displacement ellipsoids drawn at the 20% probability level. H atoms are represented as spheres of arbitrary radius. The disordered F1 and F3 atoms are shown over their two positions.

**Fig. 2.**
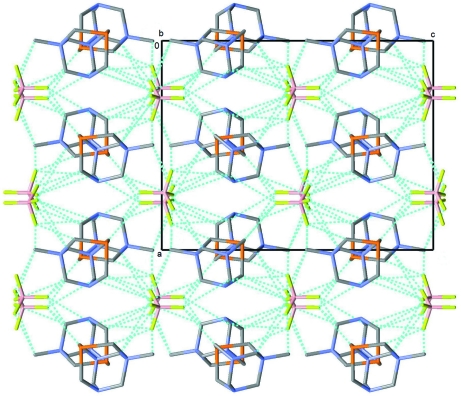
Partial representation (view along the b axis) of the crystal packing diagram of (I) showing the generation of the three-dimensional supramolecular assembly via C—H···F hydrogen bonds and C···F short contacts (blue dotted lines). Hydrogen atoms and disordered F1B and F3B atoms are omitted for clarity. C, grey; N, blue; P, orange; F, light green; B, pink.

## supplementary crystallographic information

### Comment

Being interested in the coordination chemistry of aminophosphine 1,3,5-triaza-7-phospha-adamantane (PTA) and related ligands (Kirillov *et al.*, 2007; Smoleński & Pombeiro, 2008), we have prepared compound (I) from the analogous salt, [PTA-Me]I (Daigle *et al.*, 1974; Daigle, 1998), to determine the coordination behaviour of the [PTA-Me]^+^ species in the absence of iodide ions.

Compound (I) crystallizes in an orthorhombic crystal system and its unit cell is composed of a cage-like *N*-methylated [PTA-Me]^+^ cation whose positive charge is balanced by a disordered tetrafluoroborate anion (Fig. 1). The title compound appears to be isostructural to the related salt (Jogun *et al.*, 1978) that possesses the same anion but the *P*-methylated [PTA-Me]^+^ cation. The geometrical parameters for (I) are comparable to those of other compounds with *N*-methylated PTA cores, either free (Kirillov *et al.*, 2008; Otto *et al.*, 2005; Forward *et al.*, 1996) or coordinated to the metal centres (Kovacs *et al.*, 2004; Smoleński *et al.*, 2003; Pruchnik *et al.*, 1999).

In (I), the [PTA-Me]^+^ units are disposed relatively close to the [BF_4_]^-^ anions, thus allowing their extensive assembling *via* weak intermolecular C—H···F hydrogen bonds [mean C···F separation = 3.366 (3) Å], resulting in the formation of a three-dimensional supramolecular framework (Table 1, Fig. 2).

### Experimental

An aqueous solution (25 ml) of [PTA-Me]I (1.00 mmol, 300 mg) and a methanolic (25 ml) solution of Tl[BF_4_] (1.00 mmol, 300 mg) were combined at ambient temperature [Caution: Thallium compounds are highly toxic and thus must be handled with extreme caution]. The resulting white suspension was stirred for 15 min and then filtered off, giving a white powder of thallium iodide that was discarded. The colourless filtrate was evaporated *in vacuo*, resulting in a white solid. It was recrystallized from MeOH to furnish colourless plates of (I) in *ca* 80% yield (after isolation by filtration and drying *in vacuo*).

[PTA-Me][BF_4_] is very soluble in middle-range polar solvents like Me_2_CO, CHCl_3_ and CH_2_Cl_2_, less soluble in H_2_O, MeOH, EtOH and DMSO, and insoluble in C_6_H_6_, and Et_2_O. FT–IR (KBr pellet), cm^-1^: 2965 w, 2908 w, 1461 s, 1407 s, 1347 w, 1313 s, 1292 s, 1249 s, 1120 s, 1094 s br, 1023 s, 983 s, 920 s, 899 s, 815 s, 769 s, 748 m, 732 m, 687 w, 635 w, 557 s, 534 w and 440 w. ^1^H NMR (300 MHz, D_2_O, 25°C, Me_4_Si): 4.86 and 4.75 (*J*(H^A^H^B^) = 11.4 Hz, 4H, NC*H*^A^*H*^B^N^+^), 4.52 and 4.36 (*J*(H^A^H^B^) = 13.8 Hz, 2H, NC*H*^A^*H*^B^N), 4.25 (d, ^2^*J* (H—P) = 6.8 Hz, 2H, PC*H*_2_N^+^), 3.88 and 3.75 (*J*(H^A^H^B^) = 15.3 Hz, ^3^*J*(H^A^—P) = 15.3 Hz, ^3^*J*(H^B^—P) = 8.7 Hz, 4H, PC*H*^A^*H*^B^N), 2.66 (s, 3H, N^+^C*H*_3_). ^31^P{^1^H} NMR (121.4 MHz, D_2_O, 25°C, 85% H_3_PO_4_): -85.7 (*s*).

### Refinement

All the hydrogen atoms were inserted in calculated positions (C—H = 0.98–0.99 Å) and refined as riding with *U*_iso_(H) = 1.2*U*_eq_(C) or 1.5*U*_eq_(methyl C). Atoms F1 and F3 are disordered over two sites with equal occupancies.

### Crystal data

**Table:**

C_7_H_15_N_3_P^+^·BF_4_^–^	*F*_000_ = 1072
*M**_r_* = 259.00	*D*_x_ = 1.576 Mg m^−^^3^
Orthorhombic, *P**b**c**a*	Mo *K*α radiation λ = 0.71069 Å
Hall symbol: -P 2ac 2ab	Cell parameters from 1323 reflections
*a* = 11.994 (2) Å	θ = 3.4–25.2º
*b* = 11.6933 (18) Å	µ = 0.28 mm^−^^1^
*c* = 15.569 (2) Å	*T* = 150 (2) K
*V* = 2183.5 (6) Å^3^	Plate, colourless
*Z* = 8	0.16 × 0.12 × 0.10 mm

### Data collection

**Table:**

Bruker SMART CCD diffractometer	1948 independent reflections
Radiation source: fine-focus sealed tube	1391 reflections with *I* > 2σ(*I*)
Monochromator: graphite	*R*_int_ = 0.061
*T* = 150(2) K	θ_max_ = 25.3º
ω scans	θ_min_ = 2.6º
Absorption correction: multi-scan(SADABS; Bruker, 2004)	*h* = −14→14
*T*_min_ = 0.956, *T*_max_ = 0.972	*k* = −14→13
10402 measured reflections	*l* = −18→15

### Refinement

**Table:**

Refinement on *F*^2^	Secondary atom site location: difference Fourier map
Least-squares matrix: full	Hydrogen site location: inferred from neighbouring sites
*R*[*F*^2^ > 2σ(*F*^2^)] = 0.045	H-atom parameters constrained
*wR*(*F*^2^) = 0.121	*w* = 1/[σ^2^(*F*_o_^2^) + (0.0512*P*)^2^ + 1.8936*P*] where *P* = (*F*_o_^2^ + 2*F*_c_^2^)/3
*S* = 1.05	(Δ/σ)_max_ < 0.001
1948 reflections	Δρ_max_ = 0.55 e Å^−^^3^
164 parameters	Δρ_min_ = −0.36 e Å^−^^3^
Primary atom site location: structure-invariant direct methods	Extinction correction: none

### Special details

**Table:**

Geometry. All e.s.d.'s (except the e.s.d. in the dihedral angle between two l.s. planes) are estimated using the full covariance matrix. The cell e.s.d.'s are taken into account individually in the estimation of e.s.d.'s in distances, angles and torsion angles; correlations between e.s.d.'s in cell parameters are only used when they are defined by crystal symmetry. An approximate (isotropic) treatment of cell e.s.d.'s is used for estimating e.s.d.'s involving l.s. planes.
Refinement. Refinement of *F*^2^ against ALL reflections. The weighted *R*-factor *wR* and goodness of fit *S* are based on *F*^2^, conventional *R*-factors *R* are based on *F*, with *F* set to zero for negative *F*^2^. The threshold expression of *F*^2^ > σ(*F*^2^) is used only for calculating *R*-factors(gt) *etc*. and is not relevant to the choice of reflections for refinement. *R*-factors based on *F*^2^ are statistically about twice as large as those based on *F*, and *R*- factors based on ALL data will be even larger.

### Fractional atomic coordinates and isotropic or equivalent isotropic displacement parameters (Å^2^)

**Table:**

	*x*	*y*	*z*	*U*_iso_*/*U*_eq_	Occ. (<1)
C1	0.5509 (2)	0.7943 (2)	0.68018 (19)	0.0248 (7)	
H1A	0.5329	0.8736	0.6627	0.030*	
H1B	0.6303	0.7806	0.6663	0.030*	
C2	0.3782 (2)	0.7850 (2)	0.79565 (18)	0.0245 (7)	
H2A	0.3505	0.7667	0.8539	0.029*	
H2B	0.3533	0.8635	0.7815	0.029*	
C3	0.5459 (2)	0.6251 (2)	0.80269 (18)	0.0253 (7)	
H3A	0.6251	0.6043	0.7937	0.030*	
H3B	0.5247	0.5997	0.8611	0.030*	
C4	0.5074 (2)	0.5884 (2)	0.65244 (18)	0.0219 (6)	
H4A	0.4671	0.5360	0.6133	0.026*	
H4B	0.5883	0.5749	0.6448	0.026*	
C5	0.3552 (2)	0.7325 (2)	0.64672 (18)	0.0211 (6)	
H5A	0.3364	0.8136	0.6357	0.025*	
H5B	0.3100	0.6848	0.6074	0.025*	
C6	0.3583 (2)	0.5857 (2)	0.75217 (18)	0.0216 (6)	
H6A	0.3384	0.5678	0.8124	0.026*	
H6B	0.3146	0.5344	0.7145	0.026*	
C11	0.5016 (3)	0.7311 (3)	0.53413 (18)	0.0295 (7)	
H11A	0.4557	0.6780	0.5006	0.044*	
H11B	0.5806	0.7173	0.5217	0.044*	
H11C	0.4824	0.8099	0.5187	0.044*	
B1	0.2262 (3)	0.4814 (3)	0.4904 (2)	0.0266 (8)	
N1	0.48031 (17)	0.71288 (17)	0.62773 (13)	0.0146 (5)	
N2	0.32749 (18)	0.70450 (19)	0.73373 (14)	0.0199 (5)	
N3	0.47713 (18)	0.56276 (18)	0.73947 (14)	0.0190 (5)	
F1A	0.1098 (3)	0.5043 (4)	0.4732 (3)	0.0580 (13)	0.59
F2	0.23739 (16)	0.37060 (14)	0.46049 (11)	0.0376 (5)	
F3A	0.2858 (5)	0.5568 (4)	0.4471 (3)	0.0634 (14)	0.59
F4	0.2252 (2)	0.48727 (18)	0.57697 (12)	0.0627 (7)	
P1	0.53278 (7)	0.78233 (7)	0.79717 (5)	0.0275 (2)	
F1B	0.3444 (5)	0.5214 (6)	0.4871 (5)	0.071 (2)	0.41
F3B	0.1703 (10)	0.5502 (6)	0.4431 (5)	0.096 (3)	0.41

### Atomic displacement parameters (Å^2^)

**Table:**

	*U*^11^	*U*^22^	*U*^33^	*U*^12^	*U*^13^	*U*^23^
C1	0.0204 (14)	0.0198 (15)	0.0343 (18)	−0.0049 (12)	−0.0006 (13)	0.0022 (12)
C2	0.0265 (15)	0.0223 (15)	0.0246 (16)	0.0008 (12)	0.0046 (13)	−0.0034 (12)
C3	0.0204 (15)	0.0314 (16)	0.0240 (16)	−0.0016 (12)	−0.0053 (13)	0.0056 (13)
C4	0.0266 (15)	0.0158 (14)	0.0233 (17)	0.0058 (12)	0.0027 (12)	0.0003 (11)
C5	0.0187 (14)	0.0220 (15)	0.0225 (16)	0.0024 (11)	−0.0058 (12)	0.0006 (12)
C6	0.0184 (14)	0.0244 (16)	0.0219 (15)	−0.0034 (12)	0.0007 (12)	0.0028 (12)
C11	0.0391 (17)	0.0301 (17)	0.0193 (17)	0.0029 (14)	0.0054 (13)	0.0036 (13)
B1	0.039 (2)	0.0219 (18)	0.0187 (19)	0.0019 (16)	0.0014 (15)	−0.0018 (14)
N1	0.0174 (11)	0.0138 (11)	0.0126 (12)	−0.0002 (9)	0.0006 (9)	0.0028 (8)
N2	0.0176 (11)	0.0219 (12)	0.0203 (12)	−0.0006 (9)	0.0009 (10)	0.0008 (10)
N3	0.0215 (12)	0.0179 (12)	0.0177 (13)	0.0010 (10)	−0.0011 (10)	0.0039 (9)
F1A	0.039 (2)	0.044 (3)	0.091 (4)	0.0133 (19)	−0.020 (2)	−0.007 (2)
F2	0.0468 (12)	0.0268 (10)	0.0393 (12)	0.0008 (8)	−0.0028 (9)	−0.0112 (8)
F3A	0.085 (4)	0.045 (3)	0.060 (3)	−0.027 (3)	0.034 (3)	0.009 (2)
F4	0.120 (2)	0.0407 (12)	0.0273 (12)	−0.0117 (13)	−0.0007 (12)	−0.0048 (9)
P1	0.0306 (4)	0.0273 (4)	0.0244 (5)	−0.0070 (3)	−0.0052 (3)	−0.0014 (3)
F1B	0.044 (4)	0.075 (5)	0.094 (6)	−0.032 (3)	0.031 (4)	−0.057 (4)
F3B	0.165 (10)	0.047 (5)	0.075 (6)	0.046 (6)	−0.072 (6)	−0.005 (4)

### Geometric parameters (Å, °)

**Table:**

C1—N1	1.514 (3)	C5—N1	1.547 (3)
C1—P1	1.840 (3)	C5—H5A	0.9900
C1—H1A	0.9900	C5—H5B	0.9900
C1—H1B	0.9900	C6—N3	1.463 (3)
C2—N2	1.479 (3)	C6—N2	1.466 (3)
C2—P1	1.854 (3)	C6—H6A	0.9900
C2—H2A	0.9900	C6—H6B	0.9900
C2—H2B	0.9900	C11—N1	1.495 (3)
C3—N3	1.477 (3)	C11—H11A	0.9800
C3—P1	1.847 (3)	C11—H11B	0.9800
C3—H3A	0.9900	C11—H11C	0.9800
C3—H3B	0.9900	B1—F3B	1.281 (7)
C4—N3	1.434 (3)	B1—F3A	1.319 (5)
C4—N1	1.540 (3)	B1—F4	1.350 (4)
C4—H4A	0.9900	B1—F2	1.383 (4)
C4—H4B	0.9900	B1—F1A	1.447 (5)
C5—N2	1.433 (4)	B1—F1B	1.493 (7)
			
N1—C1—P1	114.83 (18)	N1—C11—H11A	109.5
N1—C1—H1A	108.6	N1—C11—H11B	109.5
P1—C1—H1A	108.6	H11A—C11—H11B	109.5
N1—C1—H1B	108.6	N1—C11—H11C	109.5
P1—C1—H1B	108.6	H11A—C11—H11C	109.5
H1A—C1—H1B	107.5	H11B—C11—H11C	109.5
N2—C2—P1	114.14 (18)	F3B—B1—F3A	64.6 (6)
N2—C2—H2A	108.7	F3B—B1—F4	122.5 (5)
P1—C2—H2A	108.7	F3A—B1—F4	118.7 (4)
N2—C2—H2B	108.7	F3B—B1—F2	116.5 (4)
P1—C2—H2B	108.7	F3A—B1—F2	113.7 (3)
H2A—C2—H2B	107.6	F4—B1—F2	112.6 (3)
N3—C3—P1	114.38 (18)	F3B—B1—F1A	43.2 (5)
N3—C3—H3A	108.7	F3A—B1—F1A	107.7 (4)
P1—C3—H3A	108.7	F4—B1—F1A	99.6 (3)
N3—C3—H3B	108.7	F2—B1—F1A	101.8 (3)
P1—C3—H3B	108.7	F3B—B1—F1B	106.3 (7)
H3A—C3—H3B	107.6	F3A—B1—F1B	42.2 (3)
N3—C4—N1	112.3 (2)	F4—B1—F1B	91.5 (4)
N3—C4—H4A	109.1	F2—B1—F1B	101.0 (3)
N1—C4—H4A	109.1	F1A—B1—F1B	148.4 (5)
N3—C4—H4B	109.1	C11—N1—C1	109.9 (2)
N1—C4—H4B	109.1	C11—N1—C4	110.0 (2)
H4A—C4—H4B	107.9	C1—N1—C4	110.0 (2)
N2—C5—N1	111.8 (2)	C11—N1—C5	109.3 (2)
N2—C5—H5A	109.2	C1—N1—C5	110.3 (2)
N1—C5—H5A	109.2	C4—N1—C5	107.28 (19)
N2—C5—H5B	109.2	C5—N2—C6	110.1 (2)
N1—C5—H5B	109.2	C5—N2—C2	112.1 (2)
H5A—C5—H5B	107.9	C6—N2—C2	111.8 (2)
N3—C6—N2	113.1 (2)	C4—N3—C6	109.6 (2)
N3—C6—H6A	109.0	C4—N3—C3	112.6 (2)
N2—C6—H6A	109.0	C6—N3—C3	111.3 (2)
N3—C6—H6B	109.0	C1—P1—C3	96.42 (13)
N2—C6—H6B	109.0	C1—P1—C2	95.98 (13)
H6A—C6—H6B	107.8	C3—P1—C2	95.91 (13)

### Hydrogen-bond geometry (Å, °)

**Table:**

*D*—H···*A*	*D*—H	H···*A*	*D*···*A*	*D*—H···*A*
C2—H2A···F2^i^	0.99	2.54	3.438 (3)	151
C5—H5A···F4^ii^	0.99	2.35	3.314 (3)	166
C6—H6B···F4	0.99	2.46	3.364 (3)	152
C11—H11B···F2^iii^	0.98	2.43	3.350 (4)	156

Symmetry codes: (i) −*x*+1/2, −*y*+1, *z*+1/2; (ii) −*x*+1/2, *y*+1/2, *z*; (iii) −*x*+1, −*y*+1, −*z*+1.
